# Microemulsion Delivery System Improves Cellular Uptake of Genipin and Its Protective Effect against Aβ1-42-Induced PC12 Cell Cytotoxicity

**DOI:** 10.3390/pharmaceutics14030617

**Published:** 2022-03-11

**Authors:** Yujie Zheng, Guangzhi Xu, Qinxue Ni, Yan Wang, Qianxin Gao, Youzuo Zhang

**Affiliations:** 1 College of Food and Health, Zhejiang A&F University, Lin’an, Hangzhou 311300, China; zyjie@stu.zafu.edu.cn (Y.Z.); niqinxue@zafu.edu.cn (Q.N.); aliceyan@zafu.edu.cn (Y.W.); qianxingao@126.com (Q.G.); 2 Zhejiang Provincial Key Laboratory of Resources Protection and Innovation of Traditional Chinese Medicine, Zhejiang A&F University, Lin’an, Hangzhou 311300, China

**Keywords:** genipin, microemulsion, pseudo-ternary phase diagram, cellular uptake, Aβ1-42, PC12 cell

## Abstract

Genipin has attracted much attention for its hepatoprotective, anti-inflammatory, and neuroprotection activities. However, poor water solubility and active chemical properties limit its application in food and pharmaceutical industries. This article aimed to develop a lipid-based microemulsion delivery system to improve the stability and bioavailability of genipin. The excipients for a genipin microemulsion (GME) preparation were screened and a pseudo-ternary phase diagram was established. The droplet size (DS), zeta potential (ZP), polydispersity index (PDI), physical and simulated gastrointestinal digestion stability, and in vitro drug release properties were characterized. Finally, the effect of the microemulsion on its cellular uptake by Caco-2 cells and the protective effect on PC12 cells were investigated. The prepared GME had a transparent appearance with a DS of 16.17 ± 0.27 nm, ZP of −8.11 ± 0.77 mV, and PDI of 0.183 ± 0.013. It exhibited good temperature, pH, ionic strength, and simulated gastrointestinal digestion stability. The in vitro release and cellular uptake data showed that the GME had a lower release rate and better bioavailability compared with that of free genipin. Interestingly, the GME showed a significantly better protective effect against amyloid-β (Aβ1-42)-induced PC12 cell cytotoxicity than that of the unencapsulated genipin. These findings suggest that the lipid-based microemulsion delivery system could serve as a promising approach to improve the application of genipin.

## 1. Introduction

*Gardenia jasminoides* J. Ellis (also named Zhi-Zi in China), an evergreen shrub that belongs to the Rubiaceae family, is widely distributed in China and Eastern Asia. Its ripe fruit has been used as yellow natural colorants and in Chinese traditional medicine for thousands of years [1,2]. As an important Chinese traditional medicine, *G. jasminoides*’ fruit has been used to treat many different diseases due to its hepatoprotective, cholagogic, sedative, anti-hypertension, hemostasis, and detumescence properties [2]. Geniposide, an iridoid glucoside, is one of the major bioactive compounds in this medicinal plant and its content is used as the quality control marker of crude *G. jasminoides* fruit in Chinese Pharmacopeia [1].

Recently, multiple pharmacological activities of geniposide and its aglycone genipin have been reported, including its hepatoprotective effect [3], hypoglycemic [4] and anti-inflammatory activity [5], protection of cerebral ischemic injury [6], neuroprotection [7], and anti-depressant effects [8]. Although geniposide has been widely considered as the main active component, some studies suggested that genipin was a more active ingredient [9,10,11]. The pharmacokinetic studies showed that geniposide is converted into its aglycone genipin by the β-D-glucosidase of intestinal bacteria after being orally administered [12]. However, the poor water solubility is a significant barrier for genipin to cross cell membranes in the small intestine. Moreover, genipin can spontaneously react with primary ammonia compounds such as amino acids and proteins [13]. The poor water solubility and active chemical properties limit its application in food and pharmaceutical industries. Oral administration is the most common, ideal, and convenient route for many drugs or bioactive molecule administration [14]. To improve the bioavailability of genipin through oral administration, nanocrystals [15], cyclodextrin embedding [16], and hydrogel [17] were reported to improve its water solubility and intestinal absorption.

Lipid-based emulsion systems, including microemulsion, nanoemulsion, niosomes, SMEDDS (self microemulsifying drug delivery systems), and SNEDDS (self-nanoemulsifying drug delivery systems), have been proven to promote solubility and bioavailability, highlighting the potential value of this type of delivery approach for water-insoluble bioactive molecules [18,19,20]. Furthermore, encapsulating drugs in emulsion can also protect it from degeneration, control the release, and minimize the side effects [14,21].

Microemulsion is a thermodynamically stable system formed by mixing oil phase, surfactant, co-surfactant, and water phase, with a particle size of 10–200 nm [22]. The microemulsion has a small particle size and uniform dispersion, and can significantly improve the solubility of fat-soluble drugs and the stability of bioactive substances. Studies have shown that microemulsions with a specific composition can improve the oral bioavailability of poorly soluble drugs [23,24]. As a new type of drug carrier, microemulsion is widely used in a variety of clinical diseases. In clinical studies, microemulsion displays the pharmacokinetics and biodistribution of therapeutic drugs, and is able to maximize the therapeutic effects through increasing the accumulation in target tissues [25]. Accordingly, microemulsion preparations have been utilized to improve the solubility and bioavailability of various drugs, such as paclitaxel [26], quercetin [27], and lycopene [28].

Alzheimer’s disease (AD) is a neurodegenerative disease characterized by the progressive loss of memory and cognition in clinical aspects. According to epidemiological data, it is estimated that there will be more than 100 million AD patients in the world in 2050 [29]. The pathological features of AD are mainly senile plaques formed by the abnormal deposition of amyloid-β (Aβ) and neurofibrillary tangles formed by the hyperphosphorylation of tau protein. Amyloid β-protein (Aβ), which exerts neurotoxicity and synaptotoxicity, is thought to play a vital role in the pathological sequence of AD [30]. Interestingly, genipin and its derivatives have significant protective effects against Aβ-induced neurotoxicity [7,11].

In this study, genipin was encapsulated in oil-in-water microemulsion (ME) to improve the solubility and bioavailability of genipin. The stability, in vitro release, and simulated gastrointestinal digestion of genipin-containing microemulsions (GMEs) were evaluated. The cellular uptake of the GME was compared with that of free genipin using Caco-2 cells. Furthermore, the protective effect of the GME on Aβ1-42 damaged PC12 cells was investigated. Our data show that the microemulsion carrier makes genipin more resistant to gastrointestinal digestion and improves its cellular uptake and neuroprotective role.

## 2. Materials and Methods

### 2.1. Materials

Genipin (98%), medium chain triglycerides (MCT), isopropyl myristate (IPM), ethyl oleate (EO), ethoxylated hydrogenated castor oil (CO-40), labrasol, and coumarin 6 were purchased from Shanghai Yuanye Biological Technology Co, Ltd. (Shanghai, China). 3-(4,5-dimethylthiazol-2-yl)-2,5-diphenyl tetrazolium bromide (MTT), DAPI, Western and IP cell lysate kit, and a BCA protein assay kit were obtained from Beyotime Biotechnology (Shanghai, China). Dimethyl sulfoxide (DMSO), human amyloid-β (Aβ1-42, SCP0048), pepsin from porcine gastric mucosa (P7000, ≥250 units/mg), pancreatin from porcine pancreas (P7545, 8 USP), and bile salts mixture (B3426) were purchased from Sigma-Aldrich (St. Louis, MO, USA). DMEM, 0.25% trypsin, 0.02% ethylenediaminetetraacetic acid (EDTA), fetal calf serum, and phosphate buffered solution (PBS) were obtained from Gibco (Carlsbab, CA, USA). All other chemicals were analytical reagent grade.

### 2.2. Screening the Oils, Surfactants and Co-Surfactants on the Solubility of Genipin

The solubility of genipin in different oils, surfactants, or co-surfactants was investigated using the method described by Pangeni et al. [27] with some modifications. Briefly, excess genipin was mixed with 1 g of oils (soybean oil, olive oil, IPM, MCT, and EO), surfactants (Tween 20, Tween 80, Labrasol, EL-35, CO-40, and Span 80), and co-surfactants (ethanol, ethylene glycol, glycerol, and PEG 400). The mixtures were vortexed and then held at 25 ± 1.0 °C in an isothermal shaker for 24 h to allow attainment of equilibrium. After being centrifuged (10,000 rpm, 15 min), the supernatants were filtered through a 0.45 μm membrane.

The concentration of genipin was analyzed using the HPLC method as we previously described [31], with some modifications. HPLC was carried out on an Essentia LC-16 liquid chromatography system using an Ultimate XB-C18 column (250 × 4.6 mm, 10 µm, Welch Materials, Inc., Shanghai, China). After injecting 10 μL of sample, genipin was eluted isocratically with a mobile phase containing 0.15% phosphoric acid, 60% methanol, and 40% water (*v*/*v*) at a constant flow rate of 1 mL/min and detected at 238 nm. The content of genipin was calculated according to the calibration curve (peak area concentration).

### 2.3. Construction of Pseudo-Ternary Phase Diagrams and Formulation of Microemulsions

To investigate the effect of each component and the concentration on the formation of the microemulsion, the pseudo-ternary phase diagrams were constructed using the water titration method [32] at ambient temperature (25 ± 1 °C). The surfactants were blended with the co-surfactant with the ratios of 1:1, 2:1, 3:1, and 4:1 (*w*/*w*) to form the surfactant/co-surfactant mixtures (S_mix_). In each ternary phase diagram, the ratios of oil phase and surfactant/co-surfactant mixture used were 1:9, 2:8, 3:7, 4:6, 5:5, 6: 4, 7:3, 8:2, and 9:1. The total amount of oil phase and surfactant/co-surfactant mixture was 10 g. The resultant solutions were mixed by a magnetic stirrer (450 rpm) and distilled water was added in a dropwise manner until the mixtures turned transparent. The mass fraction of each component was calculated at the critical point. The oil phase, water phase, and mixed surfactant were taken as the three vertices, and a pseudo-ternary phase diagram was drawn using Origin software (version 2018) (OriginLab, Northampton, MA, USA) [27], and the area of the microemulsion area was used as the inspection index to investigate the influence of each component and ratio on the formation of the microemulsion, and determined the composition.

### 2.4. Characterization of GME

#### 2.4.1. Entrapment Efficiency (EE) and Drug Loading Efficiency (DL)

The encapsulation efficiency (EE) (%) and drug loading capacity (DL) (%) of the GME were studied by centrifugation [33]. The GMEs were transferred to an ultrafiltration centrifuge tube (MWCO 10 kDa, Millipore, Burlington, MA, USA) and centrifuged at 10,000 rpm for 15 min. The centrifuge (1 mL) was taken from the outer tube, diluted with 60% methanol aqueous solution, passed through a 0.45 μm organic filter membrane, and the free genipin measured according to the chromatographic conditions of Section 2.2. Another GME (1 mL) without centrifugation was taken, diluted with 60% methanol aqueous solution, and the total genipin content was measured according to the chromatographic conditions in Section 2.2.

The EE (%) and DL (%) were calculated using the following equations, respectively:

$$\mathrm{EE}\;(\%)\;=\;\frac{W_{\mathrm{total}\;\mathrm{drug}\;}-{\;W}_{\mathrm{free}\;\mathrm{drug}\;}}{W_{\mathrm{total}\;\mathrm{drug}}}\;\times \;100\%$$

$$\mathrm{DL}\;(\%)\;=\;\frac{W_{\mathrm{total}\;\mathrm{drug}\;}-{\;W}_{\mathrm{free}\;\mathrm{drug}}}{W_{\mathrm{Total}\;\mathrm{amount}\;\mathrm{of}\;\mathrm{GME}}}\;\times \;100\%$$

where $W_{\mathrm{total}\;\mathrm{drug}}$ is the total genipin content in GME, $W_{\mathrm{free}\;\mathrm{drug}}$ is the free genipin content after centrifugation, and $W_{\mathrm{Total}\;\mathrm{amount}\;\mathrm{of}\;\mathrm{GME}}$ is the total amount of GME, including carrier and genipin (2 mL).

#### 2.4.2. Particle Size, Polydispersity Index (PDI), Zeta Potential, and TEM Analysis

The microemulsions of genipin were analyzed in terms of mean particle size, particle size distribution (polydispersity index), and surface charge (zeta potential) by a Zetasizer (Malvern Panalytical Technologies, Malvern, UK). To avoid multiple scattering effects, the samples were diluted with 9 volume of deionized water (DI). All measurements were carried out in triplicate at a temperature of 25 ± 1 °C.

The morphology of the GME was determined by TEM. A drop of the GME was placed on a holey carbon 400 mesh copper grid. After negative staining with 2% phosphotungstic acid solution, the copper grids were dried overnight and the morphology of the GME was observed by TEM (FEI Tecnai F20, Hillsboro, OR, USA) at an operating voltage of 80 kV.

#### 2.4.3. Differential Scanning Calorimeter (DSC) Analysis

The DSC experiments were carried out using a DSC25 (TA Instruments, New Castle, DE, USA), as per the method described by Hart et al. [34], with modifications. The empty microemulsion and GME (3 mg) were added to the aluminum pans and sealed immediately. The sample was rapidly cooled to −20 °C by liquid nitrogen, and then heated to 80 °C at 10 °C/min. A blank aluminum pot was used as a reference.

### 2.5. Evaluation the Effect of Ionic Strength, Temperature and pH on GME Stability

To evaluate the effects of ionic strength on GME stability, genipin microemulsions were prepared using the method described by Chen et al. [35]. A total of 1 mL GME was mixed with 9 mL NaCl solutions to obtain the mixtures with different final NaCl concentrations (0, 100, 200, 300, 400, and 500 mM). The effect of temperature on GME stability was examined using the method described by Shi et al. [36], with a slight modification. The GMEs were placed in a water bath at different temperatures (20, 30, 40, 50, 60, and 70 °C) for 2 h. The pH stability of the GMEs was evaluated using the method previously described by Mohammed et al. [37]. The pH of the GME dispersions was adjusted with HCl or NaOH (0.1 M) to final values of 2.0, 4.0, 6.0, 8.0, 10.0, and 12.0. Ultrapure water was then added to the GME dispersions to obtain a tenfold dilution. After treatment, all samples were stored at room temperature for 24 h and the particle size, PDI, and zeta potential were measured by a Zetasizer (Malvern Panalytical Technologies, Malvern, UK), as described in Section 2.4.2.

### 2.6. Characteristics of GME during Simulated Gastrointestinal Digestion

The GME were sequentially digested in the simulated gastro fluid (SGF) and simulated intestinal fluid (SIF) to explore the fate of genipin encapsulated in microemulsions in gastrointestinal digestion, according to a protocol described previously [38]. All experiments were carried out at 37 °C in a shaker at 120 rpm and all solutions were preheated to this temperature prior to use.

For the gastric phase, simulated gastric fluid (SGF) was firstly prepared by dissolving 2 g NaCl in 1 L ultrapure water and adjusting the pH value to 2.0 ± 0.1 with 5 M HCl. Porcine pepsin was dispersed in the SGF to a final concentration of 3.2 mg/mL. The GME (2 mL) was mixed with 20 mL SGF and the mixture was incubated in the incubator shaker for 1 h at 37 °C to mimic stomach digestion.

For intestinal digestion, 20 mL of the stomach phase sample was withdrawn and the pH was adjusted to 7.0 with 1.0 M NaOH. Then, the resultant samples were mixed with simulated intestinal fluid consisting of 120 mM NaCl, 10 mM CaCl_2_, 20 mg/mL bile salts mixture, and 2 mg pancreatin, and the pH of the mixtures was readjusted to 7.0 with 0.1 M NaOH. The digestion mixtures were incubated in the incubator shaker at 37 °C for 2 h.

At end of each digestion stage, the sample was withdrawn and filtered with 0.45 µm organic filter membrane. The particle size, PDI, and zeta potential of the digested GMEs were measured by a Zetasizer (Malvern Panalytical Technologies, Malvern, UK), as described in Section 2.4.2.

### 2.7. In Vitro Release Studies

In vitro release behaviors of the genipin from GMEs and unencapsulated genipin solution were conducted using the dialysis bag method, as described by Subongkot et al. [39]. Briefly, 2 mL of each sample was transferred to the dialysis bag (with a molecular weight cut-off of 8000 Da) and dialyzed with 100 mL of aqueous hydrochloric acid (pH 1.2), ultrapure water, and PBS (pH 7.4) [40,41,42] in a beaker. To facilitate the diffusion of genipin, Tween 80 was added to all dialysis mediums to a final concentration of 0.5%. The test was maintained at 37 °C under stirring of 450 rpm. At predetermined time intervals (0.25, 0.5, 1, 2, 4, 6, 8, 10, 12, and 24 h), 1 mL of samples were taken out from the dialysis mediums and an equal volume of fresh dialysis medium was supplemented into the dialysis medium to maintain a constant volume. Eventually, all samples were then diluted with 60% methanol, filtered, and subjected to analysis by the HPLC method. Each sample was taken three times for parallel determination.

The cumulative release contents of genipin (Qn) were calculated as per the following equation:

$$Q_{n}\;=\;\left({\mathrm{WC}}_{n}+\sum_{i\;=\;1}^{n\;-\;1}C_{i}\right)/W$$

where C_n_ is the drug mass concentration measured at point n, and W is the total amount of administration.

In order to study the release kinetics of genipin, the zero-order, first-order, Higuchi, and Weibull models were employed to fit the release profiles.

Q represents the cumulative fraction of genipin released at time t.

(1) Zero-order model
  Q = a + K_0_ * t,
  where K_0_ is the zero-order release rate constant;
(2) First-order model
  Q = a * (1 − exp (−K_1_ * t)),
  where K_1_ is the first-order release rate constant;
(3) Higuchi model
  Q = K_H_ * t^1/2^ + a
  where K_H_ represents the Higuchi release rate constant;
(4) Weibull model
  Q = a * (1 − exp (−(K_W_ * (t − t_c_)) d)),
  where K_W_ represents the Weibull release rate constant.

### 2.8. Cell Culture and Cell Cytotoxic Studies

A Caco-2 human colorectal adenocarcinoma cell line and a PC12 rat adrenal pheochromocytoma cell line were purchased from the Cell Bank of the Chinese Academy of Sciences (Kunming, China). Caco-2 and PC12 cells were cultured in DMEM high glucose medium supplemented with 10% FBS, 100 U/mL penicillin, 100 μg/mL streptomycin, and 1% L-glutamine at 37 °C atmosphere containing 5% CO_2_.

The cytotoxicity of empty microemulsion, unencapsulated genipin, and GME were examined using an MTT cell viability assay before the cellular uptake experiments. Caco-2 and PC12 cells (5000 cells per well) were incubated in 96-well plates for 24 h to allow attachment. The cells were then cultured in 100 μL fresh medium containing free genipin or GMEs with various concentrations (1.25–100 μg/mL) of genipin. For the empty microemulsion group, the cells were cultured in 100 μL of fresh medium containing empty microemulsion at various concentrations (50–6000 μg/mL). After 24 h incubation, 10 μL MTT solution (5 mg/mL in PBS) was added to each well and incubated for a further 4 h at 37 °C. The MTT solution was then removed and the formed insoluble purple formazan crystals were dissolved in 150 μL DMSO. The absorbance was measured at 490 nm using a microplate reader (SpectraMax, Molecular Devices, CA, USA). The cell viability was expressed as the percent of living cells compared with the control wells.

$$\mathrm{Cell}\;\mathrm{viability}\;(\%)\;=\frac{{\mathrm{OD}}_{\mathrm{sample}}\;-{\;\mathrm{OD}}_{\mathrm{blank}}}{{\mathrm{OD}}_{\mathrm{control}}\;-{\;\mathrm{OD}}_{\mathrm{blank}}}\;\times \;100\%$$

### 2.9. Cellular Uptake Studies

Confocal laser scanning microscopy (CLSM) and HPLC were conducted to evaluate the influence of encapsulated genipin by microemulsion on the cellular uptake of genipin by Caco-2 cells. To prepare the coumarin 6-labeled GME, both coumarin 6 and genipin were dissolved in EO, and then the oil phase was mixed with surfactant/co-surfactant mixtures and the GME was formed as per the method described in Section 2.3.

For the analysis of the cellular uptake of genipin by CLSM, Caco-2 cells (2 × 10^5^ cells/well) were incubated in 12-well plates for 24 h. Subsequently, the culture media were supplemented with free coumarin 6 (C6 group), coumarin 6, and GME mixture (mix group), and coumarin 6-labeled GME (GME group), respectively. After incubation at 37 °C for 0.5, 1, 2, and 4 h, the cells were gently washed three times using PBS and fixed with 4% paraformaldehyde (*w*/*v* in PBS pH 7.2) for 15 min. The cell nuclei were stained with DAPI. The cellular uptake was examined using CLSM 510 Meta (Olympus, FV3000, Tokyo, Japan) with an oil immersion objective (40×) [43].

For the quantitative analysis of the cellular uptake of genipin, Caco-2 cells (2 × 10^5^ cells/well) were incubated in 12-well plates for 24 h. The culture media were then replaced by fresh medium supplemented with free genipin or GMEs to the final concentration of genipin 10 μg/mL. The cells were collected at 0.5, 1, 2, and 4 h and gently washed three times using PBS. The harvested cells were lysed with cell lysate for Western and IP (Beyotime Biotechnology, Shanghai, China) on ice for 15 min. After being centrifuged at 12,000 rpm for 15 min, the supernatants of cell lysate were filtered and genipin were measured by HLPC. The total protein content of the cell lysate was determined using a BCA protein assay kit. The cellular uptake of genipin was calculated and expressed as the amount of genipin (μg) per mg cell protein (μg/mg protein) [44,45].

### 2.10. Protective Effect on Aβ1-42-Induced PC12 Cell Cytotoxicity

Beta amyloid (Aβ1-42) was dissolved in DMSO to obtain a 2 mM stock solution. PC12 cells were cultured in 96-well plates at a density of 5000 cells per well. After incubation for 24 h, the cells were injured by various Aβ1-42 concentrations (2.5, 5, 10, 20, and 40 μM) for 24 h and the cell viabilities were determined using the MTT method. The proper concentration of Aβ1-42 to induce PC12 cell cytotoxicity was chosen according to the cell viability.

To determine the influence of encapsulated genipin by microemulsion on its neuroprotection effects, the protective effects of free genipin and GMEs on Aβ1-42-induced PC12 cell cytotoxicity were compared. The PC12 cells were cultured in 96-well plates at a density of 5000 cells per well for 24 h. The cultured medium was replaced with 100 μL fresh medium containing free genipin or GMEs with various genipin concentrations (1.25, 2.5, 5.0, and 10 μg/mL) for 2 h. The cells were further subjected to treatment with 20 µM Aβ1-42 for 24 h and the cell viability was examined by MTT assay. The cell viability of cells without the treatment with 20 µM Aβ1-42 was defined as 100%. 

### 2.11. Statistical Analysis

The experimental results were analyzed by one-way ANOVA with post hoc Tukey’s HSD test or Student’s *t*-test (SPSS version 24.0, IBM, Armonk, USA). All experiments were carried out in triplicate (*n* = 3), with data expressed as mean ± standard deviation (SD). The significance levels were determined using p values as indicated in the legends.

## 3. Results and Discussion

### 3.1. Solubility of Genipin in Oils, Surfactants, and Co-Surfactants

The excipients have a major role for microemulsion formation. It was revealed that excipients with the best solubilizing capability for the drug ensured the maximum drug loading and the stability of the final formulation [46]. To select the excipients, the solubility of genipin in different oils, surfactants, and co-surfactants was investigated. As presented in Table 1, genipin was more soluble in MCT, IPM, and EO than in soybean oil and olive oil. MCT, IPM, and EO were therefore chosen as the oil phases for the construction of the pseudo-ternary phase diagrams. To select the surfactant phases, the equilibrium solubility of genipin in Tween 20, Tween 80, Labrasol, EL-35, CO-40, Span 80, and Tween 80: CO-40 (1:1, *w*/*w*) was compared (Table 1). The dissolved amount of genipin in Tween 80, CO-40, Labrasol, and Tween 80: CO-40 (1:1, *w*/*w*) was higher than that of other surfactants. Therefore, Tween 80, CO-40, Labrasol, and Tween 80: CO-40 (1:1, *w*/*w*) were selected as the surfactant phases. As for the co-surfactant, the solubility of genipin in ethanol was higher than that of ethylene glycol, glycerol, and PEG 400 (Table 1). During microemulsion formation, the lower molecular weight of the co-surfactant could easily induce the opening of the tight junction of the surfactant and bring the interface film closer [25,32,35]. In addition, ethanol is a co-surfactant suitable for oral preparations, and thus was selected as the co-surfactant.

### 3.2. Construction of Pseudo-Ternary Phase Diagrams and Formulation of Microemulsions

**Figure 1:**
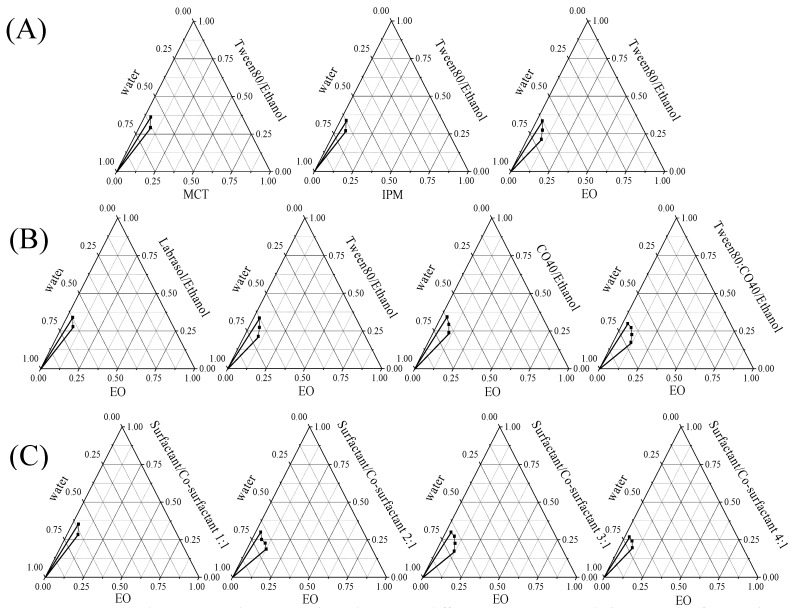
Pseudo-ternary phase diagram in different components. Oil phases (**A**), surfactant phases (**B**), and surfactant/co-surfactant ratios (**C**).

Pseudo-ternary phase diagrams were constructed to determine the excipient ratios of genipin microemulsion formation. By using Tween 80/ethanol as a surfactant/co-surfactant mixture (surfactant/co-surfactant ratio 3:1), the influence of MCT, IPM, and EO on genipin microemulsion formation was compared. As shown in Figure 1A, the microemulsion area formed by EO was the largest, indicating that EOis the most suitable oil phase for preparing the genipin microemulsion. It needs to be noted that, as one of the harmless oil phases designated by the US Food and Drug Administration, EO is well-tolerated in terms of digestion and has been widely used in topical medicines [47,48].

The effects of Tween 80, CO-40, Labrasol and Tween80: CO-40 (1:1, *w*/*w*) on genipin microemulsion formation were conducted using EO as oil phase. As shown in Figure 1B, the pseudo-ternary phase diagram area of the Tween 80/CO-40 composite surfactant was larger than that of the single surfactant, demonstrating a synergistic effect between Tween 80 and CO-40. The composite surfactant was proposed to improve the emulsification efficiency and stability of the microemulsion by reducing the molecular steric hindrance of the surfactant and increasing the flexibility of the oil–water interface [49], which may explain the better emulsification efficiency of Tween 80: CO-40 composite surfactant.

The surfactant and co-surfactant contribute to the reduction in the interfacial tension between water and oil and the proper surfactant/co-surfactant ratio is important for the stability of the microemulsion. To evaluate the influence of the Tween 80:CO-40/ethanol ratio on microemulsion formation, S_mix_ with surfactant/co-surfactant ratios of 1:1, 2:1, 3:1, and 4:1 was prepared and the microemulsion areas were measured. As shown in Figure 1C, the area of the microemulsion gradually increased along with the surfactant/co-surfactant ratio from 1:1 to 3:1. However, the microemulsion area decreased when the surfactant/co-surfactant ratio reached 4:1. Excessive ethanol and surfactants would reduce the strength and stability of the interface film by the attractive force between the surfactant head groups, which are not conducive to the formation of microemulsion [35,50]. This may be the reason for the surfactant/co-surfactant ratio of 3:1 being the best Tween 80: CO-40/ethanol ratio for microemulsion formation. While excessive surfactant may cause safety problems and increase the viscosity of the sample, the surfactant/co-surfactant ratio of 2:1 was selected here for microemulsion formation.

Based on the above pseudo-ternary phase diagram results, the GMEs were eventually developed using EO as the oil phase, Tween 80: CO-40 (surfactant)/ethanol (co-surfactant) (surfactant/co-surfactant ratio 2:1) as S_mix_, with oil: S_mix_ ratio of 9:1.

### 3.3. Characterization of Genipin-Containing Microemulsions (GME)

The encapsulation efficiency (EE) (%) and drug loading capacity (DL) (%) are the crucial parameters to evaluate the performance of microemulsion formulation. The EE and DL of the GMEs were 64.11 ± 0.58% and 3.21 ± 0.03%, respectively. The EE (%) of the GMEs was higher than that of the geniposide liposomes (44.87%) prepared with lecithin/cholesterol, but its DL (%) was lower than that of the geniposide liposomes (5.16%) [51]. Microemulsions developed by EO, Tween 80: CO-40, and ethanol can effectively embed genipin.

**Figure 2:**
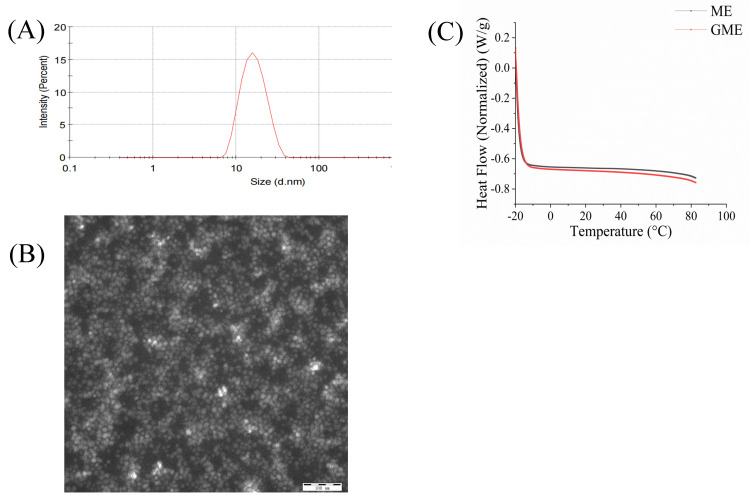
The particle size, morphology, and DSC melting thermograms of the GME. Particle size distribution (**A**); TEM (**B**); scale bar = 100 nm; DSC melting thermograms of GME and empty microemulsion (**C**).

The particle size and PDI are critical to drug release and oral absorption. The particle size and PDI of the GME were measured by a laser particle size analyzer. As shown in Figure 2A, the mean particle size was 16.17 ± 0.27 nm and the PDI was 0.183 ± 0.013. The results indicated that the GME had small droplets with homogeneous dispersibility. Its zeta potential was −8.11 ± 0.77 mV. Particles with higher absolute value of charge (negative or positive) repulse each other, which contributes to the microemulsion stability in solution [52,53]. The GME had negative zeta potentials, indicating its stability in low ionic-strength aqueous solutions. The morphology of the GME was observed by TEM. As shown in Figure 2B, the surface of the GME was smooth, quasi-spherical, and rounded in appearance. The GME was uniformly dispersed and had a narrow size distribution with an average diameter of 16.48 nm, which was consistent with the results obtained by the laser particle size analyzer.

DSC experiments were conducted to investigate the physical state of the empty microemulsion and GME. As shown in Figure 2C, the empty microemulsion and GME did not show an absorption peak with temperatures ranging from −20 °C to 80 °C, indicating that microemulsions were in an amorphous state in the formulation. A similar observation was reported by Hart et al. [34]. Here, ethanol was used as co-surfactant for the microemulsion formation. Due to the relative polarity, co-surfactants are usually distributed between the oil and surfactant tail in the microemulsion; this can reduce the interfacial tension and break liquid crystalline structures [54,55].

### 3.4. Effect of Environmental Stresses on GME Stability

Microemulsion-based delivery systems may experience a variety of environmental stresses (e.g., pH, ionic strength, or temperature changes) during storage, transportation, and utilization. Therefore, the influence of pH, ionic strength, and temperature on the physical stabilities of GMEs was examined and the results are shown in Table 2.

The GMEs were placed in a water bath at different temperatures (20, 30, 40, 50, 60, and 70 °C) for 2 h and the particle size, PDI, and zeta potential were measured. The temperature did not have a significant effect on the particle size, PDI, or zeta potential (*p* > 0.05) of the GME, in the range of 20–70 °C. In fact, the surfactants were proposed to be stable at high temperature. Moreover, heat treatment can enhance the interaction between Tween 80 and CO-40 and form a strong protective barrier [36]. Tween 80 and CO-40 may form a stable mixture with water and ethanol in GMEs, which can ensure a perfect fit at high temperatures and ensure that the entire system is in a balanced state.

In contrast, the particle size and zeta potential of the GME varied along with the increase in the pH value (*p* < 0.01) (Table 1). The mean particle size increased from 17.62 ± 0.07 nm at pH 2.0 to 28.43 ± 0.41 nm, and the zeta potential decreased from −1.50 ± 1.33 mV at pH 2.0 to −12.40 ± 0.72 mV. The phenomenon was consistent with the results found in stearic acid-based lipid nanoparticles by Ife et al. [56]. As a non-ionic surfactant, the surface charge of Tween 80 is not affected by H+ concentration, which can make the system more stable and therefore confer its stability under acidic conditions. However, the surfactant (Tween 80) and the oil phase (EO) of the microemulsion were esters, which undergo hydrolysis under alkaline conditions. With the increase of hydrolysis, the surface charge of the microemulsion changed and the distance between the surfactants increased, which reduced the repulsive force between the microemulsion particles. Therefore, as the pH increased, the microemulsion polymerized and the particle size increased [37,41,56]. The PDI of the GMEs did not vary significantly within the pH range of 2–12, indicating that it has an ideal pH stability.

To investigate the effect of ionic strength on its stabilities, the GMEs were prepared by dilution with different final concentrations of NaCl solution (0, 100, 200, 300, 400, and 500 mM) and the particle size, PDI, and zeta potential were measured. As illustrated in Table 2, the average particle size and zeta potential of the GMEs increased in a NaCl concentration-dependent manner. The average particle size of the GME under 500 mM NaCl was 41.16 ± 1.21 nm, which was more than twice the size of the GME without NaCl. The zeta potential of the GMEs increased from −7.25 ± 0.69 mV to −1.66 ± 1.78 mV with the increase of NaCl concentration from 0 to 500 mM. The influence of ionic strength on the PDI was relatively weak. The PDI increased significantly until concentrations of NaCl up to 500 mM. This may be attributed to the fact that, with the increase of NaCl concentration, the ion gradient between the inner and extra microemulsion membranes is increased, and the surface charge is reduced by the electrostatic shielding effect, which compromises the solubility of the surfactants and causes the microemulsion particles to coagulate [36,57,58]. In addition, the absorbency of electrolytes on the surface of the microemulsion can affect the hydration of the surfactant head group and cause a low surface tension, thereby exerting influence on the stability of the microemulsion [59].

In summary, although the zeta potential of the GMEs varied with pH and ionic strength, the PDI remained stable under most of the test conditions. The results indicated that the GMEs had good stability under different pH, temperature, and ionic strength environments.

### 3.5. Stability of GME in Simulated Gastrointestinal Digestion

**Figure 3:**
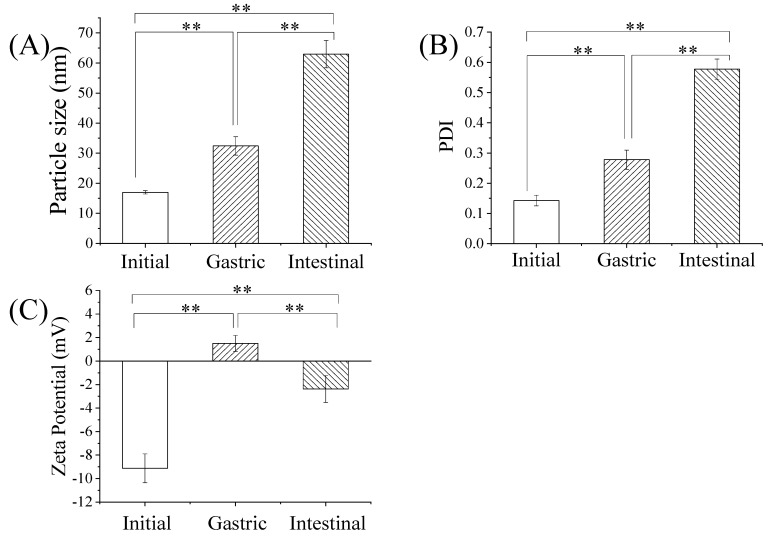
The effects of simulated gastrointestinal tract digestion on the physical characteristics of GME. Mean particle size in nm (**A**), polydispersity index (**B**), and zeta potential (mV) (**C**) of GME at the initial, gastric, and intestinal phases., ** *p* ≤ 0.01.

In order to examine whether microemulsion could improve the stability of genipin, the GMEs were digested with simulated gastrointestinal digestive juice and its particle size, PDI, and zeta potential were measured. The particle size of the GME increased from 17.01 ± 0.53 nm untreated, to 32.44 ± 3.07 nm under digestion with simulated gastric juice, and finally to 62.93 ± 4.56 nm under intestinal digestion (Figure 3A). Accordingly, the simulated gastrointestinal digestion also exerted a significant impact on the PDI of the GME, which increased from 0.143 ± 0.018 to 0.577 ± 0.034 under digestion (Figure 3B). In contrast, the zeta potential of the GME increased from −9.13± 1.22 mV to 1.49 ± 0.68 mV after simulated digestion in gastric juice, while the zeta potential was reversed to −2.38 ± 1.14 mV under simulated intestinal conditions (Figure 3C).

The results showed that the microemulsion system could significantly improve the stability of genipin under simulated gastrointestinal digestion. Genipin can directly react with primary ammonia compounds [13]. The encapsulation of genipin in microemulsion prevents the reaction with amino acids or proteins, which might enhance its bioavailability [60]. EO was reported to resist lipolysis in simulated gastrointestinal conditions [61], and the non-ionic surfactant Tween 80 remained stable in gastric conditions [62], which may contribute to the stability of the GME in simulated gastrointestinal digestion.

### 3.6. In Vitro Release Kinetics of GME

**Figure 4:**
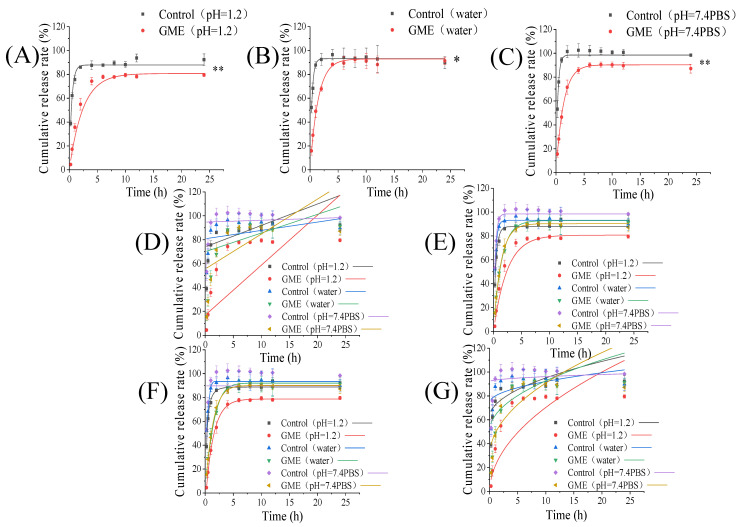
In vitro release of genipin in different dialysis media and the models’ fitting curves. pH = 1.2 aqueous hydrochloric acid (**A**), water (**B**), pH = 7.4PBS (**C**), zero-order model (**D**), first-order model (**E**), Higuchi model (**F**), and Weibull model (**G**); * *p* < 0.05, ** *p* < 0.01.

To investigate the in vitro release behaviors of genipin from GME, the GME and unencapsulated genipin were dialyzed with pH 1.2 hydrochloric acid aqueous solution, ultrapure water, and PBS (pH 7.4), respectively, and the release of genipin was measured by HPLC. Microemulsion demonstrated a significant effect on delaying the release of genipin. As shown in Figure 4A–C, the cumulative released fraction of unencapsulated genipin reached the equilibrium state at approximately 2 h, while the genipin cumulative released equilibrium state of the GME in hydrochloric acid solution, ultrapure water, and PBS was 8 h, 6 h, and 6 h, respectively. The release rate of the GME in hydrochloric acid solution (Figure 3A) showed a slow-release behavior compared with that in ultrapure water (Figure 4B) and PBS (Figure 4C). Non-ionic surfactants, like Tween 80 and CO-40, were barely affected by pH, which may render it stable under acidic conditions. On the other hand, the salt ions compete for water molecules in the solution and reduce the solubilization ability of the surfactant, which may alter the microemulsion structure [59]. This may also contribute to a delay of genipin release in PBS.

The kinetics models can be used to reveal the release mechanism of the drug from the microemulsion [63]. To gain an insight into the release mechanism of GME in different media, the zero-order, first-order, Higuchi, and Weibull models were employed to fit the GME release profiles (Figure 4D–G). The equations and correlation coefficient (R^2^) of the different models are shown in Table 3. The model with the highest correlation coefficient (R^2^) was generally considered the most suitable [64]. As can be seen in Table 3, except free genipin dialyzed with PBS, the release profiles of the other samples fitted well with the Weibull model, with the R^2^ values above 0.97942. In contrast, the first-order model fitted well with all of the in vitro release profiles of genipin, with the R^2^ values above 0.92898, indicating that the first-order model accurately describes the release mechanism as the drug release from liposomes [65]. According to the first order model, the value of the release rate constant K_1_ represented the release rate [66]. The K_1_ of the GME for dialysis with hydrochloric acid aqueous solution, ultrapure water, and PBS (pH 7.4) was 0.41, 0.70, and 0.75, respectively, which were significantly lower than that of free genipin, suggesting that encapsulation could dramatically reduce its release rate.

### 3.7. In Vitro Cellular Uptake of GME Study

**Figure 5:**
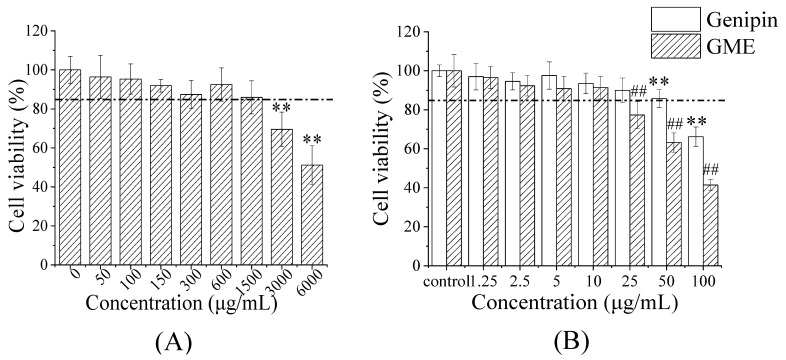
Cytotoxicity of empty microemulsion, free genipin, and GME on Caco-2 cells. Empty microemulsion (**A**); free genipin and GME (**B**). Error bars are SD (*n* = 6); ** *p* ≤ 0.01; ## *p* ≤ 0.01. Compared with the group treated with the same concentration of free genipin.

To evaluate the effect of lipid encapsulation on the genipin bioavailability, the cellular uptake was carried out using Caco-2 cells. An MTT assay was conducted first to establish the potential toxicity of the empty microemulsion, free genipin, and GME on the Caco-2 cells. The empty microemulsion did not significantly influence the cell viability within concentrations below 1500 μg/mL (Figure 5A). As shown in Figure 5B, unencapsulated genipin and GME had no significant influence on the cell viability of Caco-2 cells within the concentration range of 0–10 μg/mL. Compared with genipin, the GME did indeed have an appreciable impact on the Caco-2 cell viability when the concentrations increased up to 25 μg/mL. The cell viability of the control group was approximately 89.97% at 25 μg/mL of genipin concentration, but for GME treatment, the cell viability decreased to 77.31% at the same concentration. It has been reported that microemulsions have a dose-dependent cytotoxicity [67], which may lead to reduced cell viability at high GME concentrations. For this reason, we selected 10 μg/mL of genipin concentration to evaluate the cell uptake, ensuring that the GME did not exert a significant effect on the cell viability of Caco-2.

**Figure 6:**
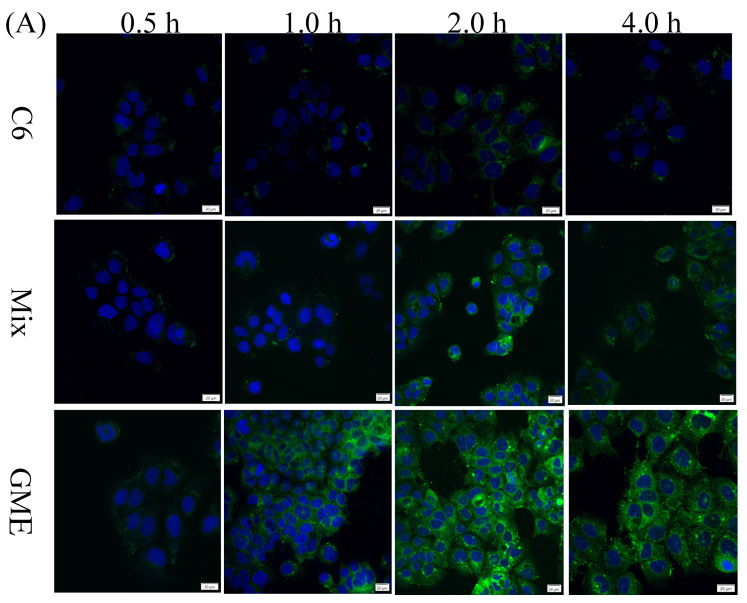

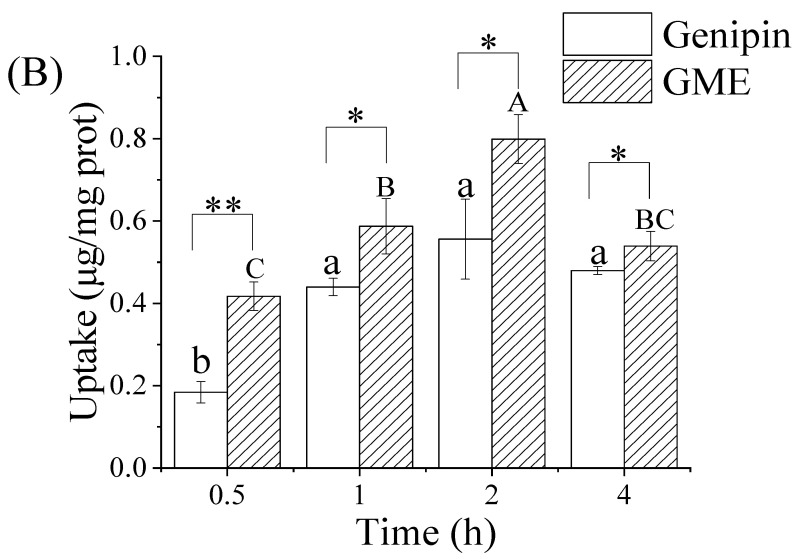
Cellular uptake of GME quantified with CLSM and HPLC. Confocal microscopy images of Caco-2 cells. Scale bar = 20 μm (**A**); HPLC quantification of genipin uptake by Caco-2 cells treated by free genipin and GME at 37 °C for 0.5, 1, 2, and 4 h (**B**). Different letters (a,b) indicate a significant difference at different time in the Genipin group; different letters (A,B,C) indicate a significant difference at different time in the GME group. * *p* < 0.05, ** *p* ≤ 0.01. Compared with the group treated with the same concentration of free genipin.

The cellular uptake of unencapsulated genipin and GME was evaluated through the Caco-2 cells. It was first investigated indirectly using CLSM. The cells were treated with free coumarin 6 (C6 group), coumarin 6 and genipin mixture (mix group), and coumarin 6-labeled GME (GME group) for 0.5 h, 1.0h, 2.0 h, and 4.0 h. As shown in Figure 6A, the coumarin 6-labeled GME group showed an obvious fluorescence signal at 1.0 h, while the fluorescence signal of the other two groups appeared at 2 h. Moreover, the fluorescence intensity of the GME (GME group) was much stronger than that of the other groups. The results indicated that microemulsion could improve the cellular uptake rate and quantity of genipin. The cellular quantities of genipin were further analyzed using HPLC. As demonstrated in Figure 6B, the intracellular accumulation of genipin in the drug-loaded microemulsion group was much higher than that of the free genipin group (*p* < 0.05) at all test times. Specifically, the Caco-2 intracellular accumulation of genipin was dependent on the incubation time within 2 h (Figure 6B), while the quantities of genipin increased at 4 h. Genipin can react spontaneously with amino acids, and chemical instability may be the reason for its decrease in concentration [13].

The findings showed that with the microemulsion system, genipin was effectively internalized into the Caco-2 cells and accumulated in the cytoplasm. This enhanced penetration may be due to the presence of surfactants, which increased the permeability of the cell membrane and was conducive to the entry of genipin. In addition, the small particle size of the GME promoted better hydrophobic interaction with the Caco-2 cell membrane. The formation of a small particle size emulsion in the cell enhanced the uptake of genipin, thereby increasing the bioavailability. The GME was an anionic nanoparticle, which can be endocytosed by interacting with the positive site of the protein in the cell membrane [68,69]. Due to the repulsive interaction with the negatively charged cell surface, genipin can be readily captured by the cell [70]. In addition, the GME was an anionic nanoparticle, which can be endocytosed by interacting with the positive site of the protein on the cell membrane, and can be captured by the cell due to the repulsive interaction with the negatively charged cell surface. For the cell lines studied, the internalization of nanoparticles was highly dependent on size. The particles were only allowed to pass through the cell membrane when the size was between 10 and 30 nm. Therefore, the small droplet size of the surfactant in the microemulsion and the amphiphilic nature of the surfactant promoted genipin diffusion and receptor-mediated endocytosis. In this study, the GME droplets were smaller than 30 nm. A similar mechanism may cause the increase of genipin uptake by cells [71].

### 3.8. Protective Effect of GME on Aβ-Induced Cytotoxicity of PC12 Cells

**Figure 7:**
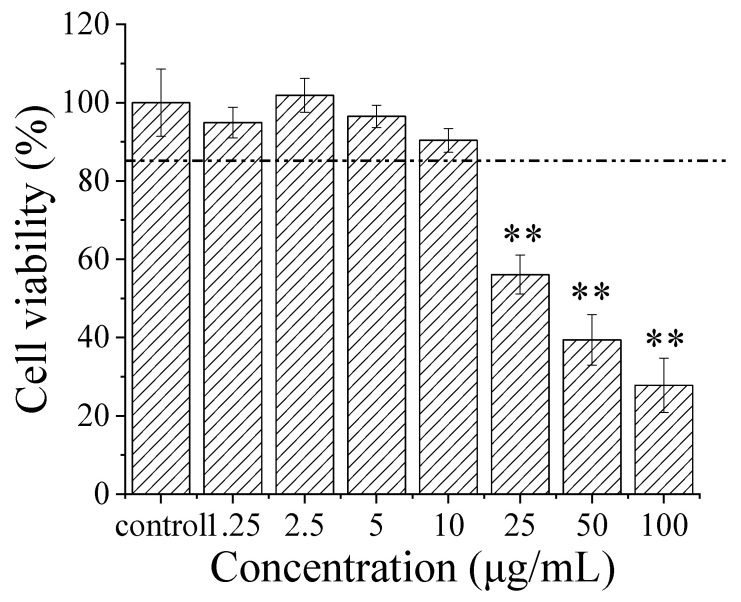
Cytotoxicity of GME on PC12 cells. Error bars are SD (*n* = 6); ** *p* ≤ 0.01. Compared with control group.

Accumulated research data show that genipin possesses therapeutic potential for central neurodegenerative diseases, such as Alzheimer’s disease (AD) and Parkinson’s disease (PD) [72]. To evaluate the influence of microemulsion on its biological activity, the protective effect of the GME on Aβ-induced PC12 cell cytotoxicity was investigated. First, the potential toxicity of the GME on PC12 was examined using an MTT assay. Although the cell viability declined with the increase in GME concentrations, the cell viability was more than 85% at the concentration of genipin within the range of 0–10 μg/mL (Figure 7). Therefore, we selected the concentration of genipin within the range of 0–10 μg/mL in our PC12 cell protection studies.

**Figure 8:**
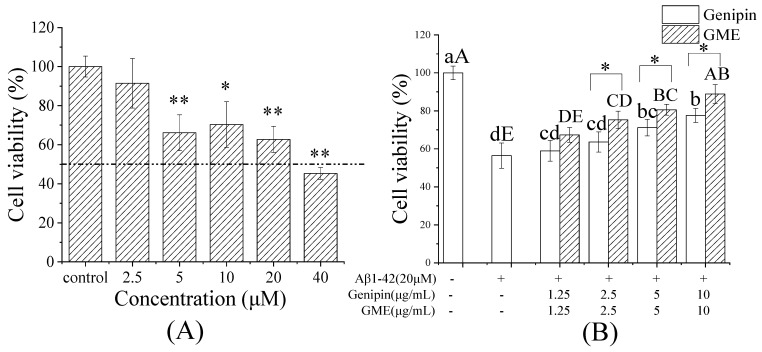
Neuroprotective effect of GME. Aβ1-42 damage concentration (**A**), and the effect of free genipin and GME (**B**). Different letters (a,b,c,d) indicate a significant difference in the Genipin group; different letters (A,B,C,D,E) indicate a significant difference in the GME group. * *p* < 0.05, ** *p* < 0.01.

To select an appropriate Aβ1-42 concentration for inducing cell damage, the PC12 cells were exposed to different concentrations of Aβ1-42 for 24 h and the cell viability was examined. As shown in Figure 8A, the survival rate of the PC12 cells declined in a Aβ1-42 dose-dependent manner. At 40 μM, the cell survival rate dropped below 50%. Considering the cells’ ability, 20 μM Aβ1-42 was used to perform the cellular uptake experiments.

To evaluate the protective effect of GMEs on Aβ-induced PC12 cell cytotoxicity, the PC12 cells were pre-protected by different concentrations of free genipin or GME (with the final concentration of genipin at 1.25, 2.5, 5, and 10 μg/mL) for 2 h, and were then treated with β-amyloid (Aβ1-42, 20 µM) for 24 h. As shown in Figure 8B, both free genipin and GMEs exhibited a protective effect for Aβ1-42-induced PC12 damage in a dose-dependent manner. As expected, the PC12 cells pre-treated with GME (2.5, 5.0, 10 μg/mL of genipin) had a significantly higher cell viability than that of free genipin (*p* < 0.05). The results indicated that the GME better protected the PC12 cells from the toxicity of Aβ1-42. These findings demonstrate that GMEs may significantly increase the cellular uptake of drugs and be an efficient delivery method for the drug treatment of CNS disorders [73].

## 4. Conclusions

In this study, genipin microemulsions (GMEs) were developed using EO as an oil phase, Tween 80: CO-40/ethanol (surfactant/co-surfactant ratio 2:1) as S_mix_, with oil: S_mix_ ratio of 9:1. The GMEs had a small size (16.17 ± 0.27 nm), with an encapsulation efficiency (EE) (%) of 64.11 ± 0.58% and demonstrating relatively high environmental (temperature, pH, and ionic strength) and simulated gastrointestinal digestion stability. GMEs significantly improve the cellular uptake rate and the protective effect on Aβ1-42-induced PC12 cell damage. These results indicate that the lipid-based microemulsion genipin delivery system could serve as a promising approach to improve its application in food and pharmaceutical industries.

## Figures and Tables

**Table 1:** Solubility of genipin in different oils, surfactants, and co-surfactants.

Phases		Solubility (mg/mL)
Oils	MCT	0.603 ± 0.007
	EO	0.570 ± 0.010
	IPM	0.446 ± 0.008
	Soybean	0.249 ± 0.017
	Olive	0.291 ± 0.006
Surfactants	Tween 20	2.494 ± 0.116
	Tween 80	3.327 ± 0.208
	Span 80	0.808 ± 0.078
	Labrasol	3.206 ± 0.064
	CO-40	2.863 ± 0.062
	EL-35	2.755 ± 0.087
	Tween80: CO-40 (1:1)	3.353 ± 0.158
Co-surfactants	Glycerol	22.29 ± 0.579
	Ethanol	36.59 ± 0.922
	PEG400	29.35 ± 0.840
	Ethylene	27.28 ± 1.175

**Table 2:** The effect of different factors on the stability of GMEs. Different letters (a,b,c,d) in the same column indicate that there is a signifcant difference between each condition group. (*p* < 0.05).

		Average Particle Size/nm	PDI	Zeta Potential/mV
Temperature/(°C)	20	16.69 ± 0.27 a	0.183 ± 0.013 a	−8.11 ± 0.77 a
	30	16.31 ± 0.35 a	0.172 ± 0.011 a	−7.86 ± 1.59 a
	40	16.52 ± 0.54 a	0.161 ± 0.022 a	−9.55 ± 0.83 a
	50	16.11 ± 0.65 a	0.175 ± 0.005 a	−6.72 ± 1.39 a
	60	16.15 ± 0.38 a	0.157 ± 0.017 a	−7.94 ± 0.90 a
	70	16.43 ± 0.38 a	0.162 ± 0.016 a	−9.41 ± 0.62 a
pH	2	17.62 ± 0.07 cd	0.135 ± 0.029 b	−1.50 ± 1.33 a
	4	17.64 ± 0.40 cd	0.153 ± 0.034 ab	−1.66 ± 0.22 a
	5.6	16.53 ± 0.60 d	0.057 ± 0.005 c	−7.25 ± 0.67 b
	6	16.77 ± 0.27 d	0.139 ± 0.015 b	−7.14 ± 0.38 b
	8	18.54 ± 0.62 c	0.192 ± 0.003 a	−10.63 ± 0.35 c
	10	22.21 ± 0.53 b	0.203 ± 0.007 a	−11.43 ± 0.68 c
	12	28.43 ± 0.41 a	0.169 ± 0.093 ab	−12.40 ± 0.72 c
NaCl concentration /(mM)	0	17.76 ± 0.27 d	0.204 ± 0.013 b	−7.25 ± 0.67 b
	100	21.01 ± 0.99 d	0.226 ± 0.023 b	−2.59 ± 0.62 a
	200	25.02 ± 0.26 c	0.220 ± 0.019 b	−3.06 ± 0.13 a
	300	27.27 ± 1.05 c	0.230 ± 0.026 b	−2.36 ± 0.33 a
	400	33.19 ± 2.02 b	0.244 ± 0.013 b	−2.01 ± 1.76 a
	500	41.36 ± 1.61 a	0.325 ± 0.019 a	−1.66 ± 1.78 a

**Table 3:** The equations and correlation coefficients (R^2^) of different release models.

Sample	Release Kinetic Models			
	Zero-Order	First-Order	Higuchi	Weibull
Genipin (pH = 1.2 hydrochloric acid)	Q = 73.95 + 1.80 × t	Q = 87.86 × (1 − exp (−2.33 × t))	Q = 10.58 × (t^(1/2)) + 61.62	Q = 88.76 × (1 − exp( − (3.55 × (t − 0.13))^0.64))
	R^2^ = 0.1672	R^2^ = 0.98524	R^2^ = 0.39335	R^2^ = 0.9889
GME (pH = 1.2 hydrochloric acid)	Q = 16.70 + 4.19 × t	Q = 80.67 × (1 – exp (−0.41 × t))	Q = 22.67 × (t^(1/2)) − 1.37	Q = 78.75 × (1 − exp( − (0.71 × (t − 0.17))^0.98))
	R^2^ = 0.59977	R^2^ = 0.99077	R^2^ = 0.84307	R^2^ = 0.99972
Genipin (water)	Q = 80.78 + 0.70 × t	Q = 93.13 × (1 – exp (−2.93 × t))	Q = 5.61 × (t^(1/2)) + 74.35	Q = 93.43 × (1 − exp( − (0.40 × (t + 2.13))^4.48))
	R^2^ = 0.00827	R^2^ = 0.96665	R^2^ = 0.17475	R^2^ = 0.97942
GME (water)	Q = 69.91 + 8.85 × t	Q = 92.91 × (1 − exp (−0.70 × t))	Q = 13.38 × (t^(1/2)) + 50.35	Q = 92.06 × (1 − exp( − (0.57 × (t + 0.25))^1.26))
	R^2^ = 0.09699	R^2^ = 0.9938	R^2^ = 0.36512	R^2^ = 0.99458
Genipin (pH = 7.4 PBS)	Q = 94.74 + 0.16 × t	Q = 98.51 × (1 − exp (−3.04 × t))	Q = 1.34 × (t^(1/2)) + 91.95	Q = 89.74 × (1 − exp( − (0.05 × (t + 40.19))^3.05))
	R^2^ = −0.02465	R^2^ = 0.92898	R^2^ = 0.05309	R^2^ = −25.8365
GME (pH = 7.4 PBS)	Q = 55.46 + 2.93 × t	Q = 90.30 × (1 − exp (−0.75 × t))	Q = 19.49 × (t^(1/2)) + 29.19	Q = 90.09 × (1 − exp( − (0.72 × (t−0.05))^1.08))
	R^2^ = 0.28428	R^2^ = 0.99861	R^2^ = 0.61602	R^2^ = 0.99834
